# Public Interest in an AI-Enabled Clinical Decision Support Tool

**DOI:** 10.1001/jamanetworkopen.2025.44672

**Published:** 2025-11-20

**Authors:** Vishal R. Patel, Michael Liu, Anupam B. Jena

**Affiliations:** 1 Harvard Medical School, Boston, Massachusetts; 2 Department of Surgery, Brigham and Women’s Hospital, Boston, Massachusetts; 3 Department of Medicine, Brigham and Women’s Hospital, Boston, Massachusetts; 4 Department of Medicine, Massachusetts General Hospital, Boston; 5 Department of Health Care Policy, Harvard Medical School, Boston, Massachusetts; 6 National Bureau of Economic Research, Cambridge, Massachusetts

## Abstract

This cross-sectional study examines internet searches and website traffic to identify recent trends in the use of artificial intelligence (AI) to aid in clinical decision-making and patient care.

## Introduction

Clinicians frequently turn to point-of-care digital support tools to guide clinical decision-making and patient care.^1,2^ Traditionally, these resources have offered evidence-based and peer-reviewed summaries of clinical topics and have been associated with improved patient outcomes.^3^ More recently, artificial intelligence (AI) platforms have emerged that generate real-time, conversational responses to clinical questions using large language models. Although AI-enabled tools have garnered widespread attention,^4^ the extent to which these resources have generated interest for clinical care is unknown.

## Methods

UpToDate is a traditional clinical resource platform that offers expert-authored summaries of clinical topics. OpenEvidence is an AI-enabled platform that generates conversational responses to point-of-care questions using large language models. The analysis focused on OpenEvidence as an a priori analysis of internet search volume across leading AI-enabled clinical reference tools (DynaMed AI and ClinicalKey AI) and showed that this AI-enabled platform accounted for 98.7% of searches during the study period. The study was exempt from consent and participant review due to the use of publicly available, deidentified data per Harvard Medical School’s institutional policy.

We analyzed internet searches and website traffic for both the traditional platform and the AI-enabled platform between January 1, 2021, and June 30, 2025. Google Trends was used to obtain relative search volumes for internet searches originating from the US for “Open Evidence” or “UpToDate” during the study period. Website traffic data for OpenEvidence and UpToDate were obtained from Semrush,^5^ which estimates site visits using clickstream data from over 200 million users.

We used Joinpoint regression to estimate average monthly percentage changes (AMPCs) in search volume and website traffic (eMethods in Supplement 1), identifying inflection points where trends shifted significantly, and pairwise tests for parallelism.^6^ To evaluate whether temporal changes in interest for the traditional platform might plausibly be associated with the availability of the AI-enabled platform, we evaluated the traditional platform’s search volumes in Canada—a neighboring country where the AI-enabled platform was not available. Hourly search trends were also examined to evaluate whether use patterns corresponded to typical clinical work hours. The analysis was performed from July 1 to August 1, 2025, using Joinpoint, version 5.2.0, and 2-tailed α levels were set at .05. The study adhered to the STROBE reporting guideline for cross-sectional studies .

## Results

**Figure 1.**
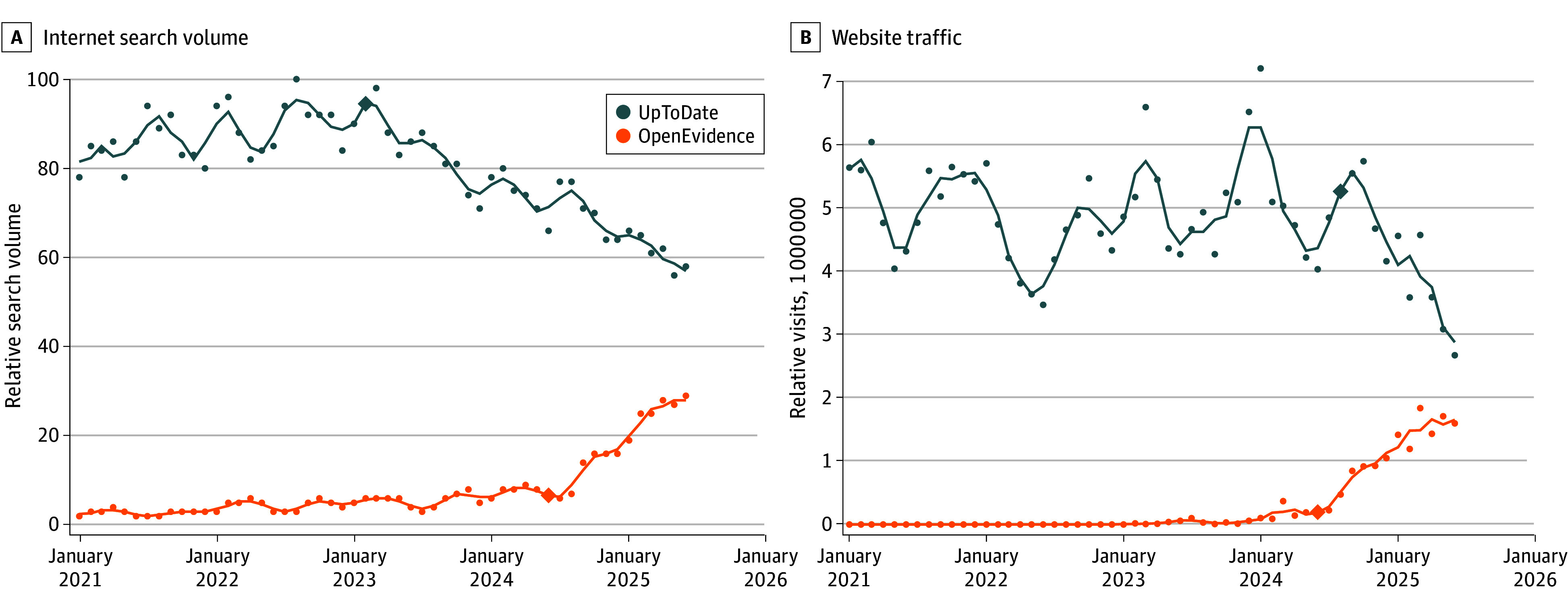
Monthly Search Volume and Website Traffic for UpToDate and OpenEvidence in the US A, Monthly internet search volumes for “UpToDate” and “OpenEvidence” from Google Trends, which reports normalized relative search interest ranging from 0 to 100 over the study period. B, displays estimated monthly website traffic to UpToDate and to OpenEvidence. Points indicate observed monthly values, the lines indicate 3-month moving averages, and the diamonds mark joinpoints for each time series. Joinpoint segments for monthly internet search volumes: UpToDate, January 2021 to February 2023 (0.4%) and March 2023 to June 2025 (−1.6%); OpenEvidence, January 2021 to June 2024 (2.8%) and July 2024 to June 2025 (13.7%). Joinpoint segments for monthly website traffic: UpToDate, January 2021 to February 2023 (0.1%) and March 2023 to June 2025 (−5.3%); OpenEvidence, January 2023 to June 2024 (28.1%) and July 2024 to June 2025 (16.9%).

Over the study period, internet search volume for the AI-enabled platform increased significantly (AMPC, 5.13% [95% CI, 4.41%-5.86%]) with a joinpoint in June 2024, whereas search volumes for the traditional platform decreased (AMPC, −0.64% [95% CI, −0.77% to −0.51%]) with a joinpoint in February 2023 (*P* < .001 for test of parallelism) (Figure 1A). Website traffic followed a similar pattern over the study period; visits to the AI-enabled platform increased from 0 to 1.59 million/month (AMPC, 19.1% [95% CI, 17.5%-20.4%]) with a joinpoint in June 2024, whereas visits to the traditional platform decreased from 5.63 to 2.67 million/month (AMPC, −1.17% [95% CI, −2.04% to −0.29%]) with a joinpoint in August 2024 (*P* < .001 for test of parallelism) (Figure 1B).

**Figure 2.**
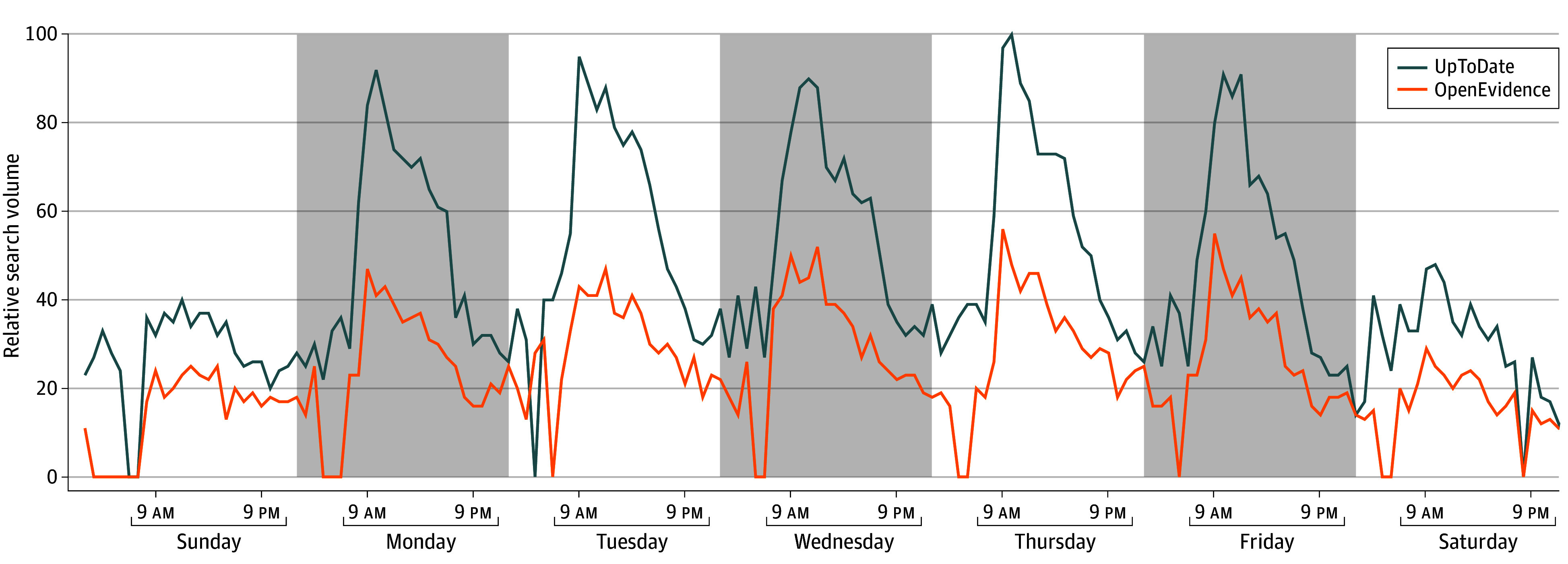
Hourly Internet Search Volume for UpToDate and OpenEvidence During the Final Week of June 2025 Hourly search interest was obtained from Google Trends, which reports normalized search volume on a scale from 0 to 100. Data are from Sunday, June 22 through Saturday, June 28, the most recent working week during the study period.

In Canada, where OpenEvidence was not available, there was no change in search interest for the AI-enabled platform over time (AMPC, −0.06% [95% CI, −0.25% to 0.14%]). Hourly search patterns during the final week of June 2025 were similar across both platforms (Figure 2).

## Discussion

In recent months, internet searches and website traffic have increased sharply for the AI-based platform relative to the traditional platform. Notably, the AI-based platform now accounts for over one-third of the traffic between the 2 platforms and approximately 1.5 million monthly visits. Limitations include the inability to determine how resources were used or user characteristics. Although traditional resources such as UpToDate have been associated with improved clinical outcomes,^3^ AI-based platforms have not yet undergone comparable rigorous validation. As health systems increasingly adopt AI-based tools in clinical care settings, it remains critical to monitor how such tools affect decision-making, health care professional experience, and patient outcomes.

## Notes

Supplement 1.
eMethods.

Supplement 2.
Data Sharing Statement

## References

[1] Del Fiol G, Workman TE, Gorman PN. Clinical questions raised by clinicians at the point of care: a systematic review. JAMA Intern Med. 2014;174(5):710-718. doi:10.1001/jamainternmed.2014.368 24663331

[2] Lott JP, Roy B, Venkatesh AK. Temporal trends in accessing online medical information. J Hosp Med. 2014;9(8):525-526. doi:10.1002/jhm.2211 24846397

[3] Isaac T, Zheng J, Jha A. Use of UpToDate and outcomes in US hospitals. J Hosp Med. 2012;7(2):85-90. doi:10.1002/jhm.944 22095750

[4] Sidoti O, McClain C. 34% of U.S. adults have used ChatGPT, about double the share in 2023. Pew Research Center. June 25, 2025. Accessed July 27, 2025. https://www.pewresearch.org/short-reads/2025/06/25/34-of-us-adults-have-used-chatgpt-about-double-the-share-in-2023/

[5] Zhukova N. Clickstream data: what is it and how does Semrush traffic & market use it? May 12, 2025. Accessed July 27, 2025. https://www.semrush.com/blog/what-is-clickstream-data

[6] Kim HJ, Fay MP, Yu B, Barrett MJ, Feuer EJ. Comparability of segmented line regression models. Biometrics. 2004;60(4):1005-1014. doi:10.1111/j.0006-341X.2004.00256.x 15606421

