# Supplementary material for: Public Interest in an AI-Enabled Clinical Decision Support Tool
Source: JAMA Netw Open. 2025 Nov 20;8(11):e2544672. doi: 10.1001/jamanetworkopen.2025.44672 (PMC12635877; doi:10.1001/jamanetworkopen.2025.44672)
Supplement: Supplement 1. — eMethods. [file jamanetwopen-e2544672-s001.pdf]

Supplemental Online Content

Patel VR, Liu M, Jena AB. Public interest in an AI-enabled clinical decision support tool. *JAMA Netw Open*. 2025;8(11):e2544672. doi:10.1001/jamanetworkopen.2025.44672

eMethods.

This supplemental material has been provided by the authors to give readers additional information about their work.

eMethods.

We conducted joinpoint regression using the Joinpoint Regression Program, version 5.4.0 (National Cancer Institute, April 2025). Analyses allowed a minimum of 0 and a maximum of 3 joinpoints. Model selection was based on permutation tests with 4,499 permutations, using a global significance level of 0.05. We specified the Poisson standard error option provided in the Joinpoint software, which estimates variability under the assumption of Poisson-distributed counts. Because website visits to www.openevidence.com were continuously zero from January 2021 through January 2023, these points were excluded from joinpoint regression, as they provided no information for slope estimation. We calculated average monthly percent changes (AMPCs) and corresponding 95% confidence intervals for each identified segment. AMPCs were derived from the slope of the regression line on the log-transformed outcome, following standard Joinpoint program procedures.
