# Supplementary material for: Public Interest in an AI-Enabled Clinical Decision Support Tool
Source: JAMA Netw Open. 2025 Nov 20;8(11):e2544672. doi: 10.1001/jamanetworkopen.2025.44672 (PMC12635877; doi:10.1001/jamanetworkopen.2025.44672)
Supplement: Supplement 2. — Data Sharing Statement [file jamanetwopen-e2544672-s002.pdf]

Data Sharing Statement

Patel. Public Interest in an AI-Enabled Clinical Decision Support Tool. *JAMA Netw Open*. Published November 20, 2025. doi:10.1001/jamanetworkopen.2025.44672

Data
Data available: No
